# Lipidomic signatures of ventilator-associated pneumonia in COVID-19 ARDS patients: a new frontier for diagnostic biomarkers

**DOI:** 10.1186/s13613-025-01492-6

**Published:** 2025-06-05

**Authors:** Arthur Kassa-Sombo, Charles Verney, Augustin Pasquet, Julien Vaidie, Deborah Brea, Virginie Vasseur, Adeline Cezard, Antoine Lefevre, Camille David, Eric Piver, Lydie Nadal-Desbarats, Patrick Emond, Hélène Blasco, Mustapha Si-Tahar, Antoine Guillon

**Affiliations:** 1 Research Center for Respiratory Diseases, INSERM U1100, University of Tours, 37044 Tours, France; 2 Intensive Care Unit, Tours University Hospital, 2 Bd Tonnellé, 37044 Tours Cedex 9, France; 3 Intensive Care Unit, Limoges University Hospital, 2 Avenue Martin Luther King, 87042 Limoges Cedex, France; 4 Imaging Brain & Neuropsychiatry iBraiN, INSERM U1253, Team Neurogenomic and Neuronal Pathophysiology, University of Tours, 37044 Tours, France; 5 Metabolomic and Biochemical Analysis Facility, US-61 ASB, INSERM, University of Tours, 37044 Tours, France; 6 Morphogenesis and Antigenicity of HIV and Hepatitis Viruses, INSERM U1259, University of Tours, 37044 Tours, France

**Keywords:** Ventilator associated pneumonia, Biomarkers, Lipidomics, Immunometabolism

## Abstract

**Background:**

Ventilator-associated pneumonia (VAP) is a significant complication in mechanically ventilated patients. Paradoxically, it lacks precise diagnostic criteria, making the identification of a reliable diagnostic indicator an unmet medical need. Lipids are critical regulators of innate lung defense. The aim of the study was to identify lipid alterations specific to VAP in tracheal aspirates of patients with ARDS.

**Methods:**

Tracheal aspirates samples from ventilated patients were collected longitudinally from patients with COVID-19-related ARDS. Tracheal aspirates sampled at the day of VAP diagnosis were used to assess VAP specific lipidome and were compared with matched controls (patients without VAP). Lipid detection was performed using ultra-high-performance liquid chromatography with high resolution mass spectrometry. The statistical analysis included: unsupervised multivariate methods, partial least squares discriminant analysis (PLS-DA), orthogonal partial least squares discriminant analysis (OPLS-DA), and the area under the receiver operating characteristic (AUROC) curve to assess classification performance. The Benjamini–Hochberg adjusted p-value was used to control the false discovery rate.

**Results:**

We studied 39 patients (26 VAP and 13 control patients). The characteristics of VAP and control patients were similar, including biological markers such as neutrophils, CRP, and PCT. The lipid signature, composed of 272 lipids, differed between VAP and control patients (p = 0.003). Phosphatidylcholines were the most represented with 17 significantly upregulated and 6 downregulated lipids. OPLSDA identified 8 best candidates as VAP biomarkers with sphingomyelin (34:1) and phosphatidylcholine (O-34:1) presenting the best scores (AUROC = 0.85 [0.71–0.95] and 0.83 [0.66–0.94], respectively). Combinations of several lipid biomarkers did not improve the prediction accuracy. During ARDS, lung lipidome mostly resulted in breakdown product of host–pathogen interactions (surfactant and pulmonary cells).

**Conclusion:**

We investigated VAP-specific lipids in tracheal aspirate and identified significant alterations in lipidomic profiles, likely driven by active infection dynamic and the breakdown of surfactant and pulmonary cells. Among the potential VAP biomarker candidates in COVID-19 ARDS, sphingomyelin (34:1) and phosphatidylcholine (O-34:1) demonstrated predictive performance for VAP that surpassed all previously tested biomarkers.

**Supplementary Information:**

The online version contains supplementary material available at 10.1186/s13613-025-01492-6.

## Introduction

Ventilator associated pneumonia (VAP) is a major complication affecting ICU patients ventilated for 48 h or more. It accounts for up to 90% of ICU-acquired infections, impacts 5–40% of ventilated patients, and drives over half of all antibiotic use in ICUs [1–4]. Despite being a major concern, VAP lacks precise definition [5–7]. VAP definition is still interpretive and poorly correlated with outcomes. It included criteria such as “a new or progressive infiltrate”, “worsening oxygenation”, and a “change in the quality or quantity of sputum”. While these criteria mirror traditional bedside diagnostic criteria for VAP, they are subjective and thus lead different physicians to come to different conclusions about whether a given patient meets criteria for VAP and eventually requires antibiotic(s) [8]. The predictive capacities for the diagnosis of VAP of serum biomarkers, e.g. procalcitonin (PCT), C-reactive protein (CRP), or soluble triggering receptor expressed on myeloid cells-1 (sTREM-1), have been investigated for decades. So far, they failed to allow adequate discrimination of VAP [9–11]. Blood leukocyte count is part of all the VAP definition criteria but also lacks specificity. The paradox where VAP is recognized as a serious disease but lacks precise diagnostic clarity complicates both clinical management and research efforts. Finding a reliable indicator for diagnosing VAP is a top priority.

The era of -omic analyses has ushered in an impressive capacity of biomarkers screening. Specifically, metabolomic analysis has recently emerged as a powerful tool for studying host–pathogen interactions, given the tights links between metabolism and inflammation [12], and represents an innovating field for biomarkers discovery [13–15]. A subset of metabolome comprises the lipidome, that is the analysis of all the lipids in a compartment. Like metabolites, lipids are closely related to inflammatory mechanisms. Lipids display a tremendous diversity with more than 500 distinct molecular species, illustrating the diverse role they play in cellular signaling [16]. Well-known examples of lipid-regulated inflammation include its initiation, driven by lipids like arachidonic acid derivatives and prostaglandins, and its resolution, mediated by phosphatidylcholines and others [17]. Very recent findings are encouraging to explore the lipidome of patients with respiratory infection: altered lipidomic profiling were observed in leukocytes and plasma of patients with community acquired pneumonia [18, 19].

We hypothesized that the lipidome differs between mechanically ventilated patients with VAP and those without VAP. Deciphering the differences may lead to biomarker discoveries. Since VAP is primarily a compartmentalized infection, we hypothesized that this analysis should be conducted at the organ level (lung) rather than the systemic level. The aim of the study is to identify lipid alterations in tracheal aspirates during VAP in patients with COVID-19-related ARDS.

## Methods

### Patients and tracheal aspirate collection

Patients (> 18-year-old) admitted to the ICU from March 21, 2021 to March 21, 2022 with positive SARS-CoV-2 RT-PCR testing and requiring invasive mechanical ventilation (MV) were prospectively included. Exclusion criteria were the presence of an initial bacterial superinfection at the time of MV initiation and previous treatment with Tocilizumab. Patients were treated according to the living WHO guideline on drugs for covid-19 [20]. Aspirates were collected during routine tracheal suctioning of mechanically ventilated patients. The biological collection was performed at the initiation of MV and subsequently every 4 days until the end of ventilation (due to death or MV weaning). Adherence to the STROBE guidelines for observational studies was maintained throughout the analysis [21]. The biological collection was approved by the local ethic committee *Espace de Réflexion Ethique de la Région Centre-Val de Loire* (DC-2014-2285). The patients or their relatives were informed about the clinical research and were able to refuse to participate to the study.

### Group assignment

Two groups were defined: control and VAP group. VAP was pragmatically defined as the initiation of antibiotic treatment for nosocomial pneumonia occurring 48 h or more after intubation, based on the treating physician’s clinical judgment, which incorporated clinical, radiological, and microbiological components of the CPIS score: body temperature, leukocyte count, tracheal secretion characteristics and volume, arterial oxygenation, chest X-ray findings, and results from tracheal aspirate cultures (Gram stain and quantitative cultures) [7]. The control group comprised patients who experienced no VAP episodes during the ICU stay.

### Sample selection

In the VAP group, tracheal aspirates were sampled on the day of VAP diagnosis and were used to assess the VAP-specific lipidome. In the control group, tracheal aspirates collected after at least 4 days of mechanical ventilation were used to match the VAP group and were analyzed as control respiratory samples. Lipididomoic alterations between the control and VAP groups were subsequently compared.

### Samples pre-analytical conditions and analysis.

Aspirates were dissociated in 3 volumes of PBS per gram, and 1 mM dithiothreitol, with stirring for 30 min. After centrifugation at 500 g for 10 min, the supernatants were stored at − 80 °C until use.

Lipid extraction steps were dissociation with isopropanol, centrifugation and dry through nitrogen flow. The residue was then reconstituted with a mix of acetonitrile, isopropanol and water.

The extraction reproducibility was evaluated by the extraction of a quality control (QC) sample obtained from a pool of 30 μL of each sample. LC-HRMS analysis was performed on a UHPLC Ultimate 3000 system (Dionex, Sunnyvale, CA), coupled to a Q-Exactive mass spectrometer (Thermo Fisher Scientific) operated in positive and negative ionization mode (ESI + /ESI-). Chromatography was carried out with a 1.7 μm EVO—C18 (150 mm × 2.10 mm × 1.7 µm) UHPLC column (Kinetex, Phenomenex, Torrance, CA) heated at 55 °C. A multistep gradient was applied over 30 min to achieve efficient lipid separation before MS analysis. At the end of the analysis, data-dependent MS/MS were performed on QC sample to annotate detected features.

### LC-HRMS data processing

Data processing was performed with Workflow4Metabolomics (W4M) using XCMS R package. Detected features were annotated according to their retention time and mass-to-charge ratio (m/z) using an in-house database built with MS/MS data. The obtained peak areas were normalized to the total signal area. Only lipids with a coefficient of variation < 30% in QC were retained to perform statistical analysis. The MS/MS spectrum of discriminant lipids were checked manually to confirm the annotation.

### Statistical analysis

For identification of lipids feature with statistically significant difference between groups, univariate and multivariate statistical analysis were employed R (version 4.2.2) MetaboAnalyst (version 6.0), GraphPad prism (version 8.0.2) and python. *P*-value of univariate analysis were computed using Mann-Withney U test. Volcano plot was used for visual representation of significant lipid alterations between the control and VAP groups. Principal Component Analysis (PCA) was employed as an unsupervised method in multivariate analysis to explore the data and uncover the directions that best explain the variance. Partial least squared discriminant analysis (PLSDA) and orthogonal partial least squared discriminant analysis (OPLSDA) were used to reduce the number of lipids in high-dimensional data and to identify spectral features that drive group separation. The variable importance in projection (VIP), and corresponding loading/contribution value in each model was used to identify the best lipids responsible to distinguish groups. The quality and performance of the multivariate OPLSDA models were assessed using the R2Y (goodness of feat parameter), Q2 (predictive ability parameter) and permutation test. For each identified lipid the difference between control and VAP groups was considered significant at Benjamini–Hochberg adjusted *p*-value < 0.05. The area under the receiver operating characteristic (AUROC) curve was computed to evaluate the classification performance.

## Results

### The lipidomic profil of tracheal aspirates is altered during VAP

**Fig. 1:**
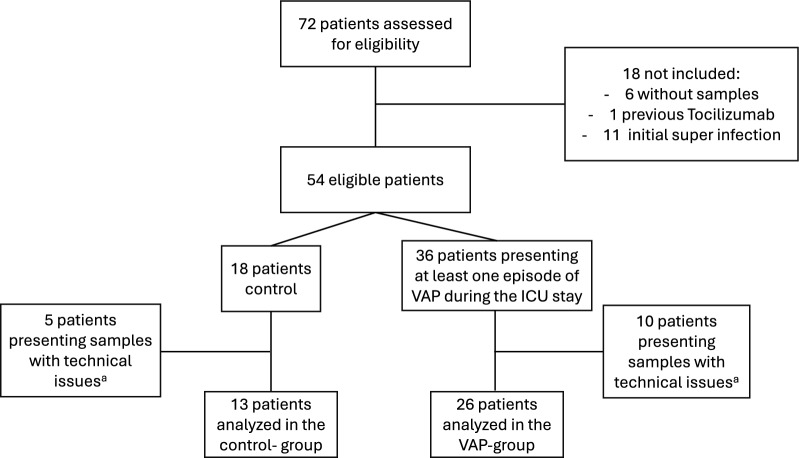
Flow chart of patients. **a** not analyzed due to technical issues (e.g., low volume and/or high viscosity)

**Fig. 2:**
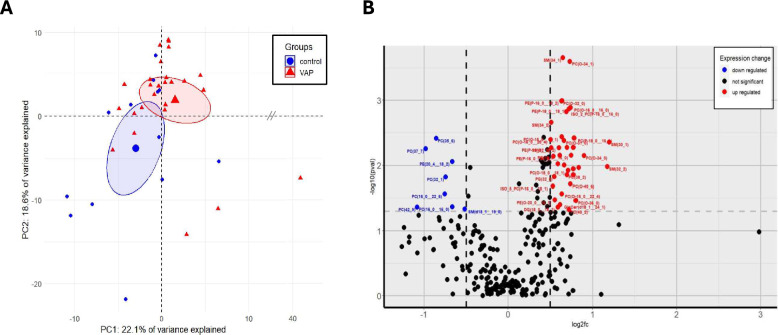
Ventilator-associated pneumonia (VAP) is associated with a major shift in pulmonary lipidome. **A** Principal component analysis of the tracheal aspirate of patients with ventilator-associated pneumonia through (VAP) or controls. PC1 was disrupted due to space limitation (full version in supplementary Fig. 2). PC1: p = 0.003. **B** Volcano plot reporting P values against fold changes. It indicates -log 10 (P-value) for lipids (Y-axis) plotted against their respective log 2 (fold change) (X-axis). The red dots represent significantly upregulated and downregulated; black indicates no significant difference

We screened 72 patients for eligibility and finally analyzed 39 patients, 26 VAP and 13 control patients (Fig. 1). Patients’ characteristics are presented in Table 1. Comorbidities and routine laboratory inflammatory biomarkers were not significantly different between groups, such as neutrophils, CRP, and PCT (respective p values 0.16; 0.40; and 0.16). Pathogens identified in VAP episodes consisted of 50% gram-positive bacteria, primarily Staphylococcus aureus, and 50% gram-negative bacteria, predominantly Enterobacteria (supplementary Fig. 1). Spectra from HRMS identified 272 lipids in the endotracheal aspiration samples. We initially employed PCA to reveal significant differences in lipidomic profiles between the control and VAP groups. The non-supervised approach exposed inherent patterns, substantiated by PCA plots (Fig. 2A and supplementary Fig. 2), confirming differences between group lipidomes (Mann–Whitney U test, p = 0.003) and explaining 40.7% of variance between groups. The PCA model demonstrated a satisfactory fit with an R2X coefficient of 0.71. The volcano plot (Fig. 2B) highlights lipids with noteworthy changes. VAP group presented more significantly upregulated lipids (n = 38) than downregulated (n = 8).

**Table 1:** Clinical characteristics

Baseline Characteristics			
	VAP patients (n = 26)	Control patients (n = 13)	p value
Characteristics			
Female sex	12 (46)	7 (54)	0.74
Age (year)	70 (62–73)	64 (59–72)	0.47
BMI (kg.m^−2^)	29.0 (24.5–31.5)	30 (25–34)	0.72
Charlson Comorbidity Index	3.0 (3.0–3.5)	3.0 (2.0–4.8)	0.48
Comorbidities			
Diabetes mellitus	3 (12)	2 (14)	> 0.99
Hypertension	18 (69)	8 (62)	0.73
COPD	3 (12)	1 (8)	> 0.99
Active smoker	1 (4)	0 (0)	> 0.99
Chronic cardiac failure	0 (0)	1 (8)	0.33
Chronic renal failure	2 (8)	2 (15)	0.59
Neoplasia (past 5-year)	1 (4)	2 (15)	0.25
Clinical features			
SOFA at sample collection	3 (3–5)	3 (3–4)	0.33
SAPS II at admission	27 (21–33)	21 (18–24)	0.08
*Respiratory parameters*			
Invasive ventilation	26 (100)	13 (100)	–
PaO2/FiO2 (mmHg)	162 (142–180)	176 (144–203)	0.23
Driving pressure	12.0 (11.0–14.0)	12.0 (11.0–13.5)	0.46
Routine laboratory measures			
Lymphocytes (G/L)	0.59 (0.35–0.80)	0.58 (0.48–0.76)	0.60
Neutrophils (G/L)	9.1 (5.1–11.6)	6.5 (5.5–9.8)	0.78
CRP (mg/L)	127 (67–193)	99 (59–115)	0.23
PCT (µg/L)	0.10 (0.10–0.50)	0.10 (0.10–0.43)	0.63

Characteristics at sample collection			
	VAP patients (n = 26)	Control patients (n = 13)	p value
Clinical features			
SOFA	3 (3–4)	3 (2–5)	0.81
Respiratory parameters			
Invasive ventilation	26 (100)	13 (100)	–
PaO2/FiO2 (mmHg)	154 (126–173)	189 (153–244)	0.06
Driving pressure	13.0 (12.0–16.0)	12.5 (12.0–14.5)	0.47
Routine laboratory measures			
Lymphocytes (G/L)	0.78 (0.47–1.09)	0.62 (0.48–1.02)	0.66
Neutrophils (G/L)	10.8 (9.0–13.4)	9.1 (7.5–10.9)	0.16
CRP (mg/L)	122 (67–165)	86 (33–151)	0.40
PCT (µg/L)	0.10 (0.10–0.35)	0.10 (0.10–0.40)	0.16

*BMI* Body Mass Index, *COPD* Chronic Obstructive Pulmonary Disease, *SOFA* Sequential Organ Failure Assessment, *SAPS II* Simplified Acute Physiology Score II, *PaO2/FiO*2 Partial arterial oxygen pressure on Fraction inspired of oxygen ratio, *CRP* serum C-Reactive Protein, *PCT* serum Procalcitonin

### Lipid landscape changes in tracheal aspirate of patients with VAP

**Fig. 3:**
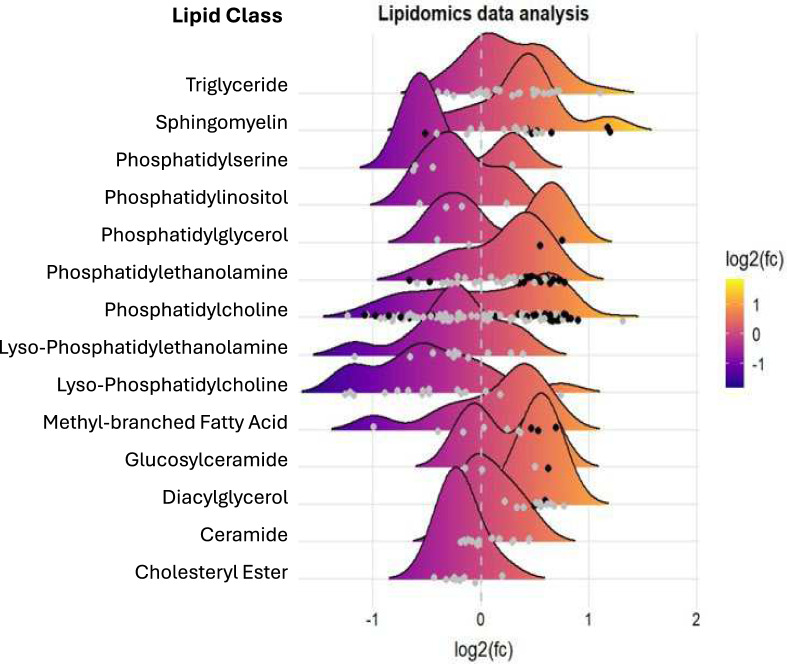
Pulmonary lipid landscape comparing patient with VAP to control. Each dot represents a lipid species. Which are grouped per lipid class. The color of the dots indicates whether the lipid was significantly different (black) between the groups. The x-axis shows the log2 fold change between groups for each lipid. The y axis indicates the different lipid classes. With a ridge plot per lipid class that shows the distribution of the lipids within their respective classes

To provide a holistic view of the lipidome, we grouped all lipids based on their respective classes and constructed a lipid landscape plot to compare tracheal aspirate from VAP and controls (Fig. 3). Among the most modified lipid family, phosphatidylcholines (PC) were the most represented with 17 upregulated and 6 downregulated lipids. Phosphatidylethanolamine (PE) and sphingomyelin (SM) represented respectively 8 and 6 variables in the top 46 dysregulated lipids by VAP.

### Identification of discriminant lipids for VAP

**Fig. 4:**
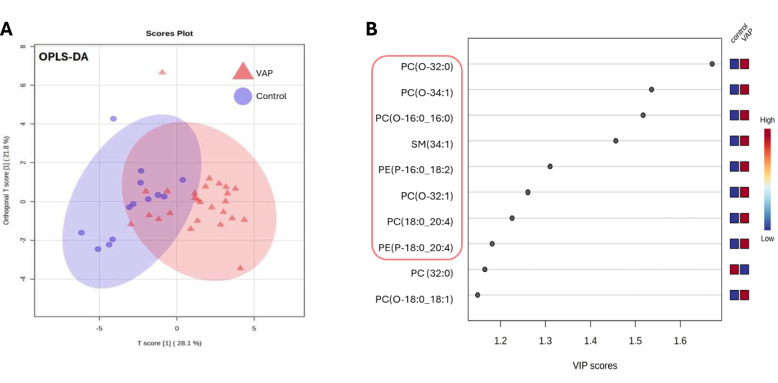
Selection of the most discriminant lipids for VAP. **A** Lipidomic profile using OPLS-DA. Internal validation of the corresponding OPLS-DA model by permutation analysis (n = 1000); fraction of the variance of descriptor class response (Y) (R2Y) = 0.573 (Green bar). p-value = 0.003; fraction of the variance predicted (cross-validated) (Q2) = 0.289 (Red bar). p-value < 0.001. **B** Variable importance in the projection (VIP) score plot showing the contribution of variables of the model. The color boxes indicate whether factor was rising or falling in the groups

To improve the lipid selection, we employed a systematic approach involving multiple steps. First, we identified the top contributive lipids from the PCA analysis (supplementary Fig. 3A). Second, we used PLS-DA and identified lipids with loading values exceeding 0.1 (supplementary Fig. 3B). PLS-DA performance assessed by quality assessment statistics reached Q2Y = 0.75 (supplementary Fig. 3C). Finally, we fitted an OPLS-DA model to the 31 selected lipids identified from the previous steps, thus explaining 49.9% of between group variance (Fig. 4A). This approach facilitated a robust selection of the most relevant lipids for studying discriminant lipid pattern changes during VAP. The OPLS-DA model demonstrated data fitting (R2Y = 0.573; p-value = 0.003) and the predictive capability (Q2 = 0.289; *p*-value < 0.001) (supplementary Fig. 4). The VIP values from OPLS-DA models were employed for feature selection (Fig. 4B). We prioritized most significant lipids based on multiple criteria: (i) inclusion in at least two different models (PCA, PLS-DA, or OPLS-DA), (ii) Mann–Whitney test adjusted p-value < 0.05, and (iii) VIP > 1 in the OPLS-DA analysis. The best 8 VIP score candidates (all upregulated in VAP) were further explored as potential VAP biomarker: SM(34:1), PE(O-18:0_20:4), PE(P-16:0_18:2), PC(O-32:0), PC(O-18:0_20:4), PC (O-34:1), PC(O-16:0_16:0), PC(O-32:1).

### Identification of the lipid candidates for VAP biomarkers

**Fig. 5:**
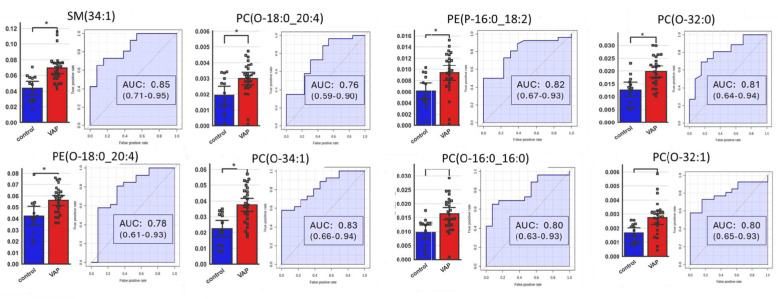
Analysis of the receiver operating characteristic (ROC) curve of potential lipids for diagnostic between patients with VAP and control. ROC curve analysis and bar plot of lipids with the highest contribution of separating the studied groups. Bar plot in blue and red colors representing control and VAP patients respectively. AUC: area under the receiver operating characteristic curve

For a direct comparison of the levels of the 8 lipids, we employed Mann–Whitney U analysis to identify critical lipids between the VAP and control groups. We compared the endotracheal lipid profiles of the two groups using the Benjamini–Hochberg method for p-value adjustment (Fig. 5). The analysis revealed that all selected lipids are significant (adjusted p-value < 0.05). ROC curves for lipids previously selected unveiled efficient AUROC values ranging from 0.78, to 0.85, the most discriminant lipids being SM(34:1) with AUROC = 0.85 (0.71–0.95) and PC(O-34:1) with AUROC = 0.83 (0.66–0.94) (Fig. 5). These AUROC values were higher than those of CRP 0.57(0.32–0.83) or PCT 0.52 (0.26–0.79) (supplementary Fig. 5). Of note, SM(34:1) and PC(O:34–1) were measured at the same abundance at the time of intubation for both control and VAP groups but had distinct kinetics thereafter (increase of both lipid markers in VAPs and decrease of both markers in controls; supplementary Fig. 6).

**Fig. 6:**
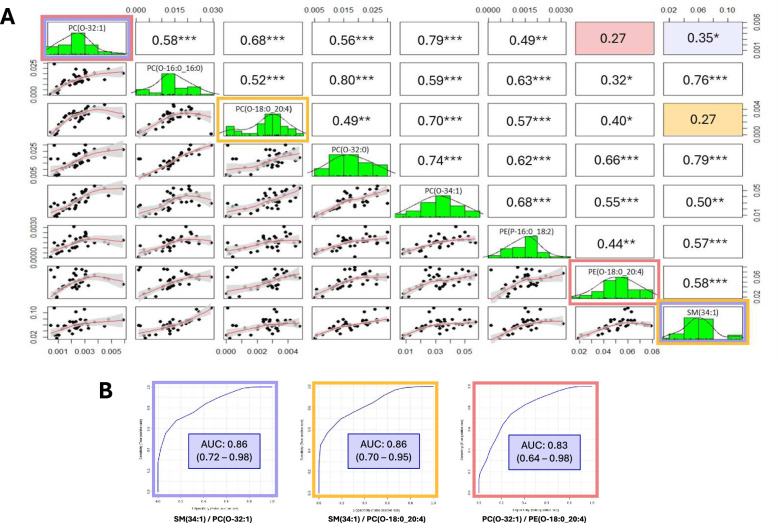
Combination of lipid biomarkers for the VAP prediction. **A** Correlation between the 8 prominent lipids: (i) the histogram of the kernel density estimation and distribution of each variable is shown on the diagonal. (ii) On the bottom of the diagonal: the bivariate scatter plots with a fitted line are displayed. (iii) On the top of the diagonal: the value of the Pearson’s correlation plus the significance level as stars; each significance level is associated to a symbol: p-values (0.001. 0.01. 0.05) relate to symbols (“***”. “**”. “*”). **B** Combination of lipid biomarkers with the lowest correlation to assess the performance of VAP prediction. The blue, yellow and red color reminders highlight the combinations used in panel (**A**)

Next, we investigated whether combining several lipid biomarkers could improve the prediction accuracy. By combining several lipid biomarkers, each representing different aspects of the disease (e.g., inflammation, infection, and membrane disruption), the prediction model may become more accurate and reliable. This hypothesis aligns with the principle that the collective value of complementary biomarkers is greater than the sum of their parts. We selected lipids that were weakly correlated to improve their discriminative capacity (Fig. 6A). These combinations moderately improved AUROC from ROC curves with the best value of 0.86 (95% CI: 0.72–0.98) obtained by combining SM(34:1) and PC(O-32:1) (Fig. 6B).

### Assessment of tracheal lipidome origin

**Fig. 7:**
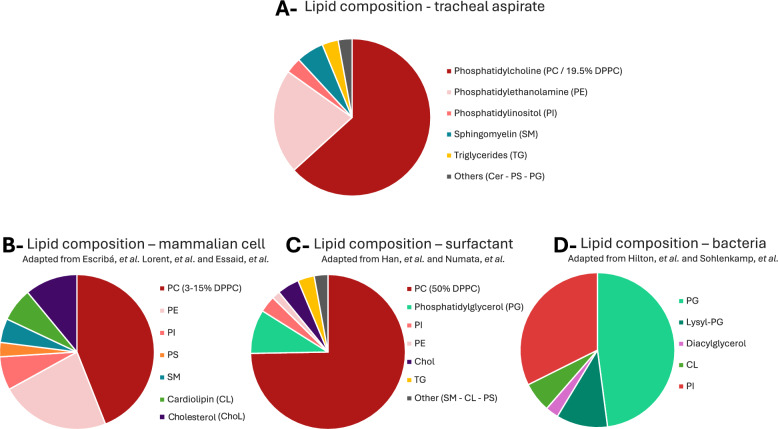
Representative proportions of lipid families found in: **A** Our pool of tracheal aspirates, **B** Mamalian cells, adapted from [30–32], **C** Human surfactant, adapted from [27, 33], **D** Bacteria species represented in our panel of VAP pathogens, adapted from [34, 35], the mean lipid proportion of all the studies on one bacteria type have been calculated and adjusted to the frequency in our VAP samples (Data missing for H.influenza). *Cer* Ceramide, *Chol* Cholesterol, *CL* Cardiolipin, *DPPC* Dipalmitoil-phosphatidylcholine, *PC* Phosphatidylcholine, *PE* Phosphatidylethanolamine, *PG* Phsophatidylglycerol, *PI* Phosphatidylinositol, *PS* Phosphatidylserine, *SM* Sphingomyelin, *TG* Triglyceride, *VAP* Ventilator Associated Pneumonia

To better understand the origin of the lipids found in tracheal aspirates, we compared its composition to lipidomes of potential sources of lipids. Representative proportions of lipid families were obtained from available published data [16–22]. We observed that the tracheal aspirate lipidome appears to be a mixture of lipids from mammalian cells and surfactant, distinct from the lipid composition of bacteria. Figure 7A summarizes the lipid family composition of tracheal aspirates (from our data) and the lipid composition of surfactant (Fig. 7B), human cells (Fig. 7C) and bacteria consistent with pathogens identified in our study (Fig. 7D).

## Discussion

Our study demonstrates significant alterations in the lipidomic profiles of tracheal aspirates during VAP in COVID-19 ARDS patients. Among the 272 detectable lipids, we identified eight that were strongly associated with the occurrence of VAP, with AUROC values indicating diagnostic performance ranging from 0.76 to 0.85. These biomarkers demonstrated superior performance compared to classical clinical and biological markers, such as neutrophils, CRP, and PCT, which did not show significant differences between VAP and control patients in our cohort.

This study is the first to perform a comprehensive analysis of lipidomic profiles in tracheal aspirates. Previous research has highlighted the compartmentalization of inflammatory mediators in the lungs, underscoring the importance of studying the host response at the site of infection rather than relying on blood samples, despite the ease of obtaining the latter [22]. In summary, two types of respiratory fluids can be collected from mechanically ventilated patients: bronchoalveolar lavage (BAL) fluid and tracheal aspirates, each with its own advantages and limitations. BAL fluid is easier to analyze but requires an invasive procedure for collection. In contrast, tracheal aspirates are routinely obtained during daily tracheal suctioning in ventilated patients and are typically considered biological waste. However, their high viscosity makes analysis more challenging. We previously established the pre-analytical conditions for immune exploration of tracheal aspirates [15, 23] and have now demonstrated the feasibility of lipidomic profiling in these samples. In our cohort, the sphingomyelin “SM(34:1)” and the phosphatidylcholines “PC(O-34:1)” showed the best performance in VAP prediction with AUROC of 0.85 and 0.83 respectively. By comparison, serum procalcitonin levels on the day that VAP was clinically suspected had an AUROC of 0.51 (95% CI 0.39–0.63) [9]. The slope of CRP from day 1 to day 6 was associated with the development of VAP, showing a predictive performance with an AUROC of 0.71 (95% CI [0.60–0.82]) [24]. The concentrations of sTREM-1 in BAL fluid did not correlate with VAP status (AUROC of 0.57) [11]. Our results may pave the way for designing assays to measure VAP-specific lipids in endotracheal aspirate in addition to microbiological exploration.

Interestingly, our finding reveals that the tracheal aspirate lipidome appears to be a mixture of lipids from mammalian cells and surfactant, distinct from the lipid composition of bacteria. It is likely that the lipids present in these respiratory samples originate from an active infection dynamic resulting in breakdown of surfactant and pulmonary cells. Phosphatidylcholine and sphingomyelin (the two main VAP-specific lipids of our cohort) play vital roles in innate immunity. They act as structural components of immune cell membranes, precursors for bioactive lipids involved in inflammation, and participants in host–pathogen interactions [25–27].

Our study has strengths and limitations. We observed differences in lipidomic signatures, despite all studied patients having similar severity (as assessed by SAPS II and SOFA scores), being initially hospitalized for a SARS-CoV-2 pneumoniae, and requiring mechanical ventilation for ARDS. While a comparison with non-infected ICU patients or outpatient controls could have helped identify altered lipids, it would have reduced the clinical relevance of such findings. The VAP group exhibited a wide variety of pathogens, with an equal proportion of Gram-negative and Gram-positive bacteria, suggesting that the findings are applicable to most nosocomial bacteria. The sample size did not allow lipidomic analyses to be stratified according to bacterial etiologies of VAP. COVID-19 pandemic resulted in increased incidences of VAP [28, 29]. This context has facilitated the collection of tracheal aspirate; the association of SM(34:1), PC(O-34:1) or PC(O-32:1) with VAP should be confirmed in absence of COVID-19. As with all research on VAP, the absence of a clear gold standard for diagnosis poses challenges [5]. No retrospective adjudication committee was used for the following reasons: (i) the single-site nature of the study; (ii) the lipid analyses were conducted blindly, without knowledge of the group definitions; (iii) we preferred a pragmatic approach, relying on the site-based diagnosis made by the medical team responsible for the patient’s care, rather than a retrospective central adjudication. Finally, confirmation cohorts are needed; our results should be considered as proof-of-concept so far.

In conclusion, our study is pioneer in investigating VAP-specific lipids in tracheal aspirate, a convenient, and low-risk biological fluid. In COVID-19 ARDS patients, we identified significant alterations in lipidomic profiles, likely driven by host–pathogen interactions and the breakdown of surfactant and pulmonary cells. Among the potential VAP biomarker candidates, sphingomyelin (34:1) and phosphatidylcholine (O-34:1) demonstrated predictive performance for VAP that surpassed all previously tested biomarkers.

## Supplementary Information

Supplementary material 1.

## Data Availability

Restrictions apply to the availability of these data and so are not publicly available. However, data are available from the authors upon reasonable request and with the permission of the institution.

## Abbreviations

AUROC: Area under the receiver operating characteristic
CAP: Community acquired pneumonia
ICU: Intensive care unit
HRMS: High resolution mass spectrometry
MV: Mechanical ventilation
OPLSDA: Orthogonal partial least squared discriminant analysis
PLSDA: Partial least squared discriminant analysis
PC: Phosphatidylcholine
PE: Phosphatidylethanolamine
PCA: Principal component analysis
ROC curve: Receiver operating characteristic curve
SM: Sphingomyelin
UHLPC: Ultra-high performance liquid chromatography
VAP: Ventilator associated pneumonia
VIP: Variable importance in projection

## References

[1] Papazian L, Klompas M, Luyt CE. Ventilator-associated pneumonia in adults: a narrative review. Intensive Care Med. 2020;46(5):888–906. 10.1007/s00134-020-05980-0. 32157357 10.1007/s00134-020-05980-0 PMC7095206

[2] Elliott D, Elliott R, Burrell A, et al. Incidence of ventilator-associated pneumonia in Australasian intensive care units: use of a consensus-developed clinical surveillance checklist in a multisite prospective audit. BMJ Open. 2015. 10.1136/bmjopen-2015-008924. 26515685 10.1136/bmjopen-2015-008924 PMC4636654

[3] Ranzani OT, Niederman MS, Torres A. Ventilator-associated pneumonia. Intensive Care Med. 2022;48(9):1222–6. 10.1007/s00134-022-06773-3. 35771252 10.1007/s00134-022-06773-3 PMC9245883

[4] Howroyd F, Chacko C, MacDuff A, et al. Ventilator-associated pneumonia: pathobiological heterogeneity and diagnostic challenges. Nat Commun. 2024;15(1):6447. 10.1038/s41467-024-50805-z. 39085269 10.1038/s41467-024-50805-z PMC11291905

[5] Torres A, Niederman MS, Chastre J, et al. International ERS/ESICM/ESCMID/ALAT guidelines for the management of hospital-acquired pneumonia and ventilator-associated pneumonia: Guidelines for the management of hospital-acquired pneumonia (HAP)/ventilator-associated pneumonia (VAP) of the European Respiratory Society (ERS), European Society of Intensive Care Medicine (ESICM), European Society of Clinical Microbiology and Infectious Diseases (ESCMID) and Asociación Latinoamericana del Tórax (ALAT). Eur Respir J. 2017;50(3):1700582. 10.1183/13993003.00582-2017. 28890434 10.1183/13993003.00582-2017

[6] Martin-Loeches I, Rodriguez AH, Torres A. New guidelines for hospital-acquired pneumonia/ventilator-associated pneumonia: USA vs Europe. Curr Opin Crit Care. 2018;24(5):347–52. 10.1097/MCC.0000000000000535. 30063491 10.1097/MCC.0000000000000535

[7] Pugin J, Auckenthaler R, Mili N, Janssens JP, Lew PD, Suter PM. Diagnosis of ventilator-associated pneumonia by bacteriologic analysis of bronchoscopic and nonbronchoscopic “blind” bronchoalveolar lavage fluid. Am Rev Respir Dis. 1991;143(5 Pt 1):1121–9. 10.1164/ajrccm/143.5_Pt_1.1121. 2024824 10.1164/ajrccm/143.5_Pt_1.1121

[8] Klompas M. Interobserver variability in ventilator-associated pneumonia surveillance. Am J Infect Control. 2010;38(3):237–9. 10.1016/j.ajic.2009.10.003. 20171757 10.1016/j.ajic.2009.10.003

[9] Luyt CE, Combes A, Reynaud C, et al. Usefulness of procalcitonin for the diagnosis of ventilator-associated pneumonia. Intensive Care Med. 2008;34(8):1434–40. 10.1007/s00134-008-1112-x. 18421435 10.1007/s00134-008-1112-x

[10] Coelho L, Rabello L, Salluh J, et al. C-reactive protein and procalcitonin profile in ventilator-associated lower respiratory infections. J Crit Care. 2018;48:385–9. 10.1016/j.jcrc.2018.09.036. 30308469 10.1016/j.jcrc.2018.09.036

[11] Palazzo SJ, Simpson TA, Simmons JM, Schnapp LM. Soluble triggering receptor expressed on myeloid cells-1 (sTREM-1) as a diagnostic marker of ventilator-associated pneumonia. Respir Care. 2012;57(12):2052–8. 10.4187/respcare.01703. 22613763 10.4187/respcare.01703 PMC4432465

[12] Tandon P, Abrams ND, Carrick DM, et al. Metabolic Regulation of inflammation and its resolution: current status, clinical needs, challenges, and opportunities. J Immunol Baltim Md 1950. 2021;207(11):2625–30. 10.4049/jimmunol.2100829. 10.4049/jimmunol.2100829 PMC9996538 34810268

[13] Lee CH, Banoei MM, Ansari M, et al. Using a targeted metabolomics approach to explore differences in ARDS associated with COVID-19 compared to ARDS caused by H1N1 influenza and bacterial pneumonia. Crit Care Lond Engl. 2024;28(1):63. 10.1186/s13054-024-04843-0. 10.1186/s13054-024-04843-0 PMC10900651 38414082

[14] Blasco H, Bessy C, Plantier L, et al. The specific metabolome profiling of patients infected by SARS-COV-2 supports the key role of tryptophan-nicotinamide pathway and cytosine metabolism. Sci Rep. 2020;10(1):16824. 10.1038/s41598-020-73966-5. 33033346 10.1038/s41598-020-73966-5 PMC7544910

[15] Guillon A, Brea-Diakite D, Cezard A, et al. Host succinate inhibits influenza virus infection through succinylation and nuclear retention of the viral nucleoprotein. EMBO J. 2022;41(12): e108306. 10.15252/embj.2021108306. 35506364 10.15252/embj.2021108306 PMC9194747

[16] Quehenberger O, Armando AM, Brown AH, et al. Lipidomics reveals a remarkable diversity of lipids in human plasma. J Lipid Res. 2010;51(11):3299–305. 10.1194/jlr.M009449. 20671299 10.1194/jlr.M009449 PMC2952570

[17] Bennett M, Gilroy DW. Lipid Mediators in Inflammation. Microbiol Spectr. 2016. 10.1128/microbiolspec.mchd-0035-2016. 27837747 10.1128/microbiolspec.MCHD-0035-2016

[18] Chouchane O, Schuurman AR, Reijnders TDY, et al. The plasma lipidomic landscape in patients with sepsis due to community-acquired pneumonia. Am J Respir Crit Care Med. 2024;209(8):973–86. 10.1164/rccm.202308-1321OC. 38240721 10.1164/rccm.202308-1321OC PMC12039242

[19] Schuurman AR, Chouchane O, Butler JM, et al. The shifting lipidomic landscape of blood monocytes and neutrophils during pneumonia. JCI Insight. 2024;9(4): e164400. 10.1172/jci.insight.164400. 38385743 10.1172/jci.insight.164400 PMC10967382

[20] Agarwal A, Hunt B, Stegemann M, et al. A living WHO guideline on drugs for covid-19. BMJ. 2020;370: m3379. 10.1136/bmj.m3379. 32887691 10.1136/bmj.m3379

[21] von Elm E, Altman DG, Egger M, et al. The Strengthening the Reporting of Observational Studies in Epidemiology (STROBE) statement: guidelines for reporting observational studies. J Clin Epidemiol. 2008;61(4):344–9. 10.1016/j.jclinepi.2007.11.008. 18313558 10.1016/j.jclinepi.2007.11.008

[22] Bendib I, Beldi-Ferchiou A, Schlemmer F, et al. Alveolar compartmentalization of inflammatory and immune cell biomarkers in pneumonia-related ARDS. Crit Care. 2021;25:23. 10.1186/s13054-020-03427-y. 33422148 10.1186/s13054-020-03427-y PMC7794625

[23] Jouan Y, Guillon A, Gonzalez L, et al. Phenotypical and functional alteration of unconventional T cells in severe COVID-19 patients. J Exp Med. 2020;217(12): e20200872. 10.1084/jem.20200872. 32886755 10.1084/jem.20200872 PMC7472174

[24] Póvoa P, Martin-Loeches I, Ramirez P, et al. Biomarker kinetics in the prediction of VAP diagnosis: results from the BioVAP study. Ann Intensive Care. 2016;6(1):32. 10.1186/s13613-016-0134-8. 27076187 10.1186/s13613-016-0134-8 PMC4830786

[25] Lee M, Lee SY, Bae YS. Functional roles of sphingolipids in immunity and their implication in disease. Exp Mol Med. 2023;55(6):1110–30. 10.1038/s12276-023-01018-9. 37258585 10.1038/s12276-023-01018-9 PMC10318102

[26] Ma J, Gulbins E, Edwards MJ, Caldwell CC, Fraunholz M, Becker KA. Staphylococcus aureus α-toxin induces inflammatory cytokines via lysosomal acid sphingomyelinase and ceramides. Cell Physiol Biochem Int J Exp Cell Physiol Biochem Pharmacol. 2017;43(6):2170–84. 10.1159/000484296. 10.1159/000484296 29069651

[27] Han S, Mallampalli RK. The role of surfactant in lung disease and host defense against pulmonary infections. Ann Am Thorac Soc. 2015;12(5):765–74. 10.1513/AnnalsATS.201411-507FR. 25742123 10.1513/AnnalsATS.201411-507FR PMC4418337

[28] Wicky PH, Dupuis C, Cerf C, et al. Ventilator-associated pneumonia in COVID-19 patients admitted in intensive care units: relapse, therapeutic failure and attributable mortality—a multicentric observational study from the OutcomeRea network. J Clin Med. 2023;12(4):1298. 10.3390/jcm12041298. 36835834 10.3390/jcm12041298 PMC9961155

[29] Boyd S, Nseir S, Rodriguez A, Martin-Loeches I. Ventilator-associated pneumonia in critically ill patients with COVID-19 infection: a narrative review. ERJ Open Res. 2022;8(3):00046–2022. 10.1183/23120541.00046-2022. 35891621 10.1183/23120541.00046-2022 PMC9080287

[30] Escribá PV, Busquets X, Inokuchi JI, et al. Membrane lipid therapy: Modulation of the cell membrane composition and structure as a molecular base for drug discovery and new disease treatment. Prog Lipid Res. 2015;59:38–53. 10.1016/j.plipres.2015.04.003. 25969421 10.1016/j.plipres.2015.04.003

[31] Lorent JH, Levental KR, Ganesan L, et al. Plasma membranes are asymmetric in lipid unsaturation, packing and protein shape. Nat Chem Biol. 2020;16(6):644–52. 10.1038/s41589-020-0529-6. 32367017 10.1038/s41589-020-0529-6 PMC7246138

[32] Essaid D, Rosilio V, Daghildjian K, et al. Artificial plasma membrane models based on lipidomic profiling. Biochim Biophys Acta. 2016;1858(11):2725–36. 10.1016/j.bbamem.2016.07.010. 27457703 10.1016/j.bbamem.2016.07.010

[33] Numata M, Voelker DR. Anti-inflammatory and anti-viral actions of anionic pulmonary surfactant phospholipids. Biochim Biophys Acta Mol Cell Biol Lipids. 2022;1867(6):159139. 10.1016/j.bbalip.2022.159139. 35240310 10.1016/j.bbalip.2022.159139 PMC9050941

[34] Sohlenkamp C, Geiger O. Bacterial membrane lipids: diversity in structures and pathways. FEMS Microbiol Rev. 2016;40(1):133–59. 10.1093/femsre/fuv008. 25862689 10.1093/femsre/fuv008

[35] Hilton KLF, Manwani C, Boles JE, et al. The phospholipid membrane compositions of bacterial cells, cancer cell lines and biological samples from cancer patients. Chem Sci. 2021;12(40):13273–82. 10.1039/d1sc03597e. 34777745 10.1039/d1sc03597e PMC8529332

