# Supplementary material for: Lipidomic signatures of ventilator-associated pneumonia in COVID-19 ARDS patients: a new frontier for diagnostic biomarkers
Source: Ann Intensive Care. 2025 Jun 5;15:78. doi: 10.1186/s13613-025-01492-6 (PMC12141186; doi:10.1186/s13613-025-01492-6)

Supplementary figure 1.

Proportion of identified bacterias in VAP diagnostics, n=28 bacteria in 26 VAP episodes.

MSSA: Methicillin Susceptible Staphylococcus Aureus, MRSA: Methicillin Resistant Staphylococcus Aureus,

GNB: Gram Negative Bacteria (*Pseudomonas aeruginosa* and *Stenotrophomonas maltophila*), H.influenzae: *Haemophilus influenzae*

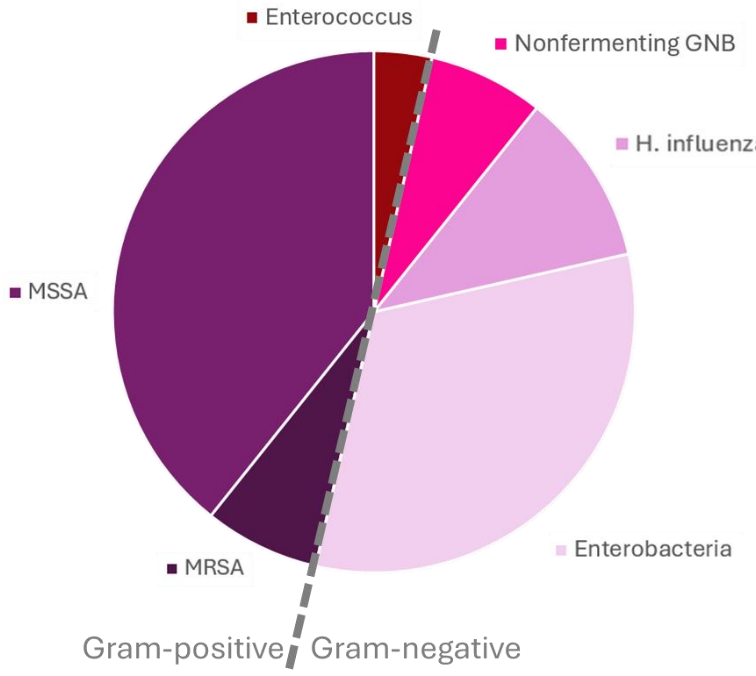

Supplementary Figure 2.

Principal component analysis of the tracheal aspirate of patients with ventilator-associated pneumonia through (VAP) or controls (without disruption of PC1).

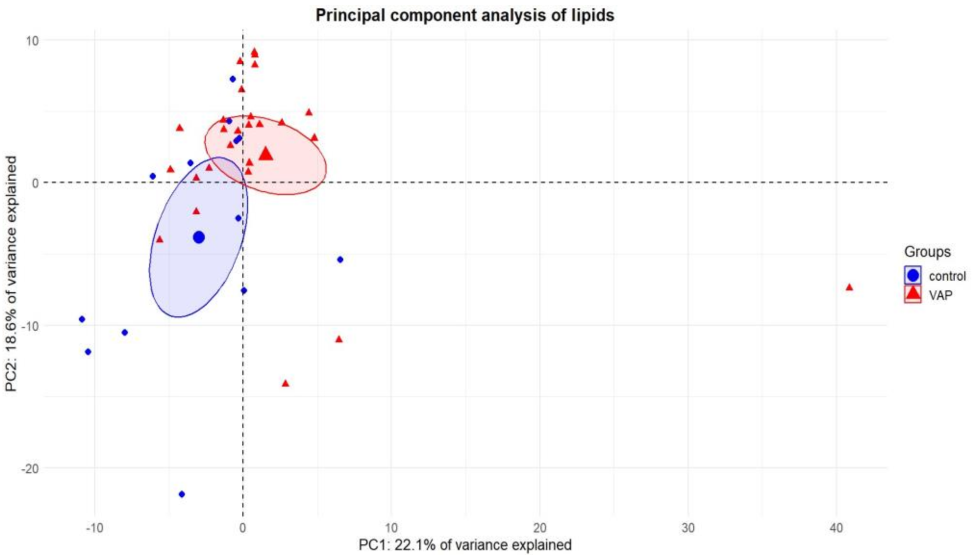

Supplementary Figure 3.

A- Lipids contribution to the first PCA component; B- PLSDA score plot; C- Loadings plot

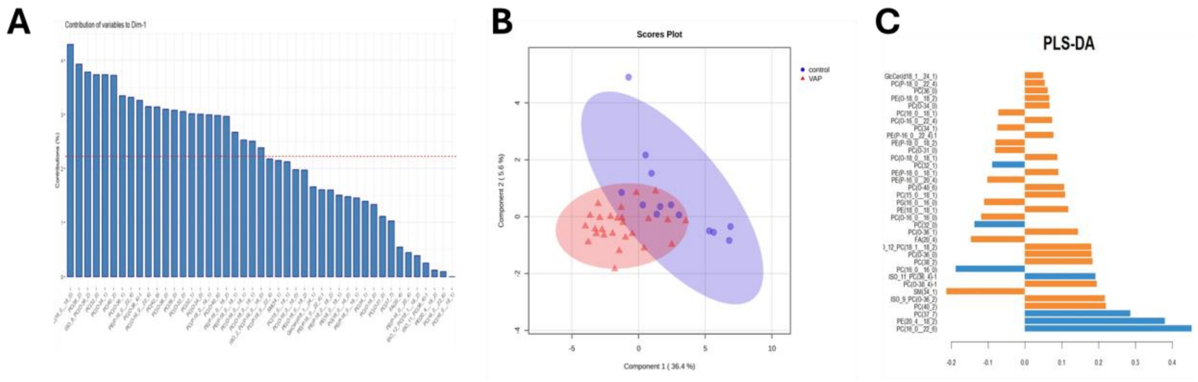

Supplementary Figure 4.

Internal validation of the corresponding OPLS-DA model by permutation analysis (n = 1000); fraction of the variance of descriptor class response (Y) (R2Y) = 0.573 (Green bar). p-value =0.003; fraction of the variance predicted (cross-validated)(Q2) = 0.289 (Red bar). p-value < 0.001.

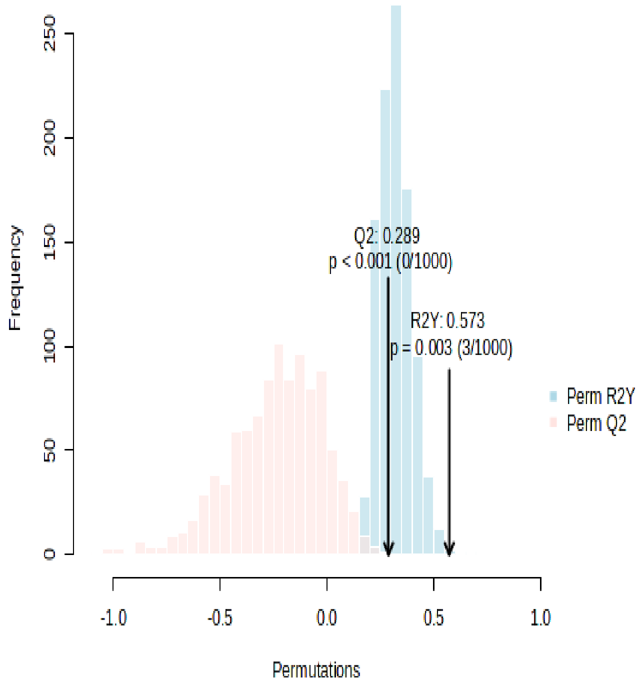

Supplementary Figure 5.

ROC cures and AUC values of CRP (A) and PCT (B) as biomarkers for diagnosis of VAP.

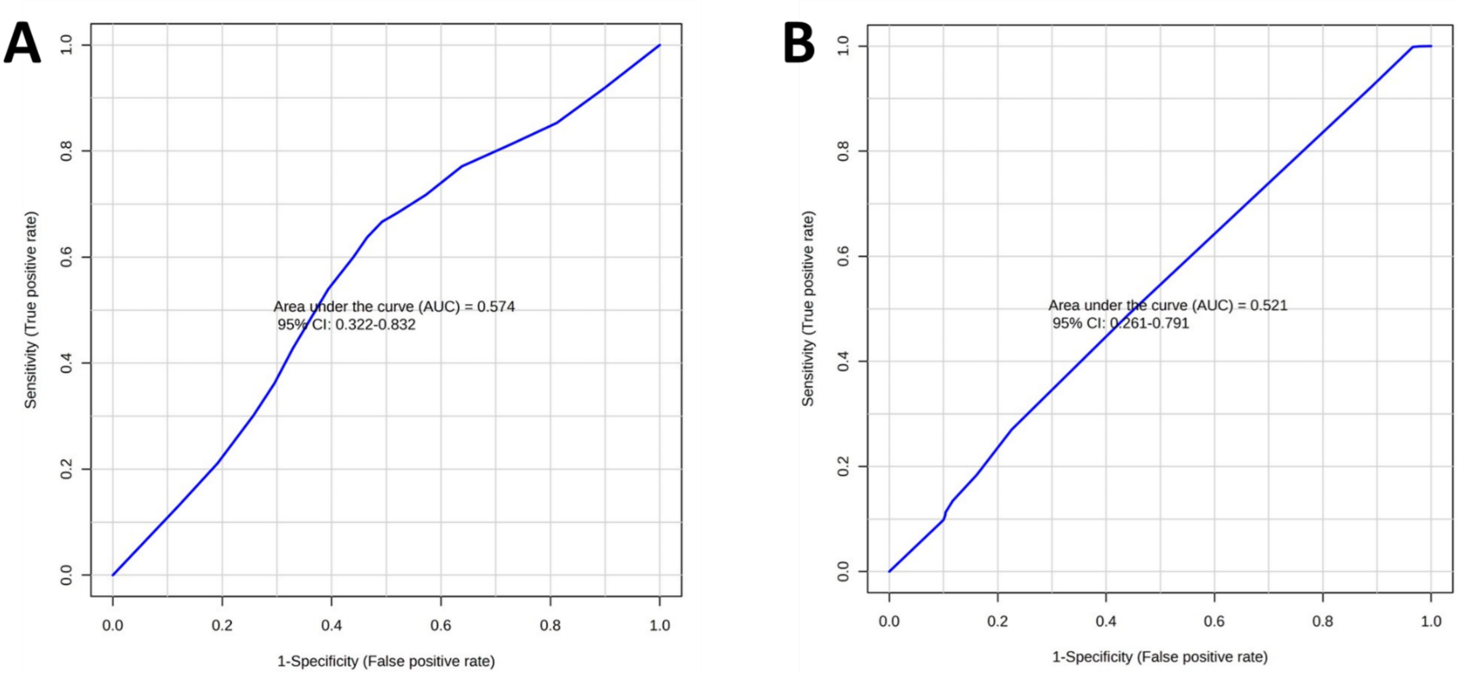

Supplementary Figure 6.

Semiquantitative dosages of SM(34:1) for panel A-, and PC(O-34:1) for panel B-, in control and VAP group, in the tracheal aspirate collected at baseline (the day of intubation). Evolution of the dosages of SM(34:1) for panel C-, and PC(O-34:1) for panel D-, from intubation and over time in tracheal aspirates, performed every 4 days. Mann Whitney test used at every time point.

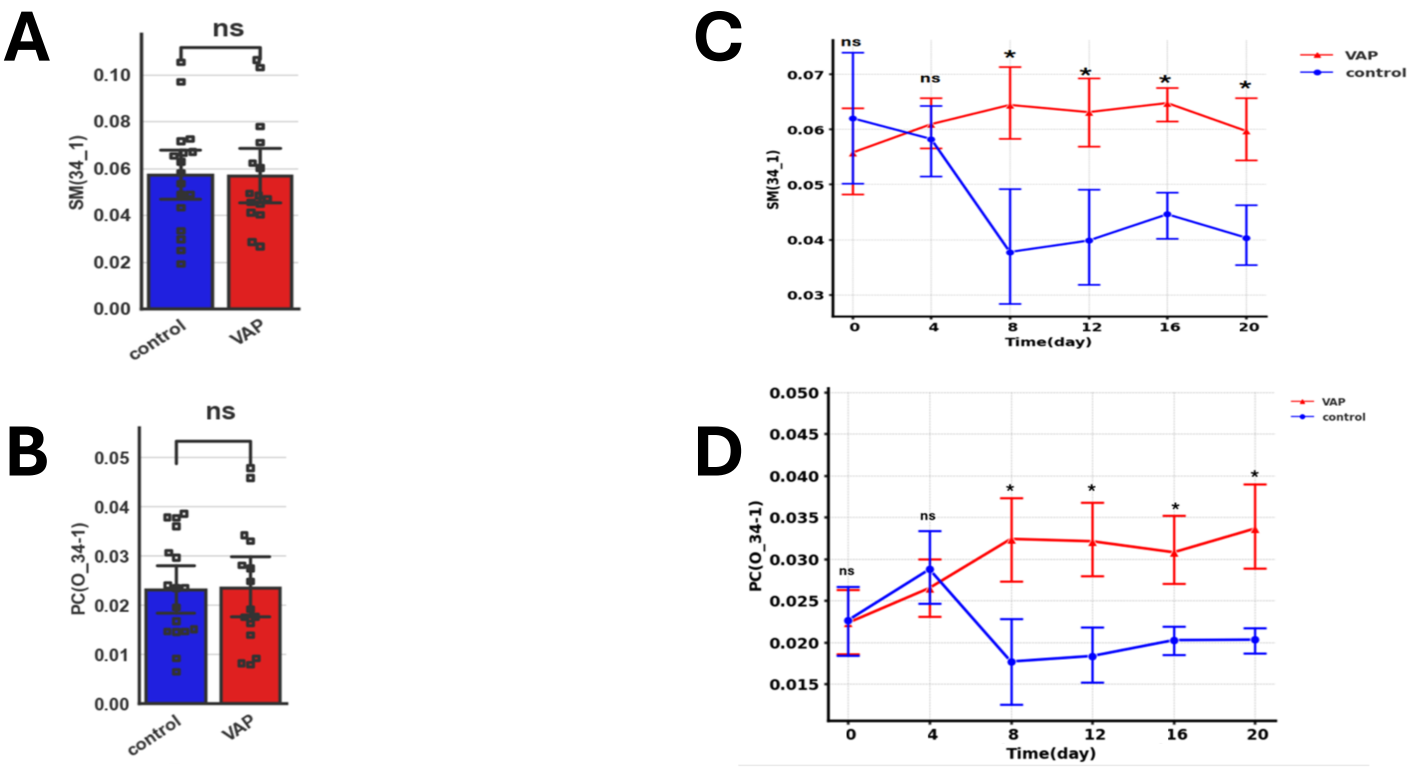

Supplement: Supplementary file 1 — Supplementary material 1. [file 13613_2025_1492_MOESM1_ESM.pdf]
